# Voluntary liquorice ingestion increases blood pressure via increased volume load, elevated peripheral arterial resistance, and decreased aortic compliance

**DOI:** 10.1038/s41598-017-11468-7

**Published:** 2017-09-08

**Authors:** Elina J. Hautaniemi, Anna M. Tahvanainen, Jenni K. Koskela, Antti J. Tikkakoski, Mika Kähönen, Marko Uitto, Kalle Sipilä, Onni Niemelä, Jukka Mustonen, Ilkka H. Pörsti

**Affiliations:** 1 Faculty of Medicine and Life Sciences, FIN-33014 University of Tampere, Tampere, Finland; 2 Nutrition Unit, Tampere University Hospital, Tampere, 33521 Finland; 3 Department of Internal Medicine, Tampere University Hospital, Tampere, 33521 Finland; 4 Department of Clinical Physiology, Tampere University Hospital, Tampere, 33521 Finland; 5 Department of Laboratory Medicine and Medical Research Unit, Seinäjoki Central Hospital, Seinäjoki, 60220 Finland

## Abstract

We investigated the haemodynamic effects of two-week liquorice exposure (glycyrrhizin dose 290–370 mg/day) in 22 healthy volunteers during orthostatic challenge. Haemodynamics were recorded during passive 10-minute head-up tilt using radial pulse wave analysis, whole-body impedance cardiography, and spectral analysis of heart rate variability. Thirty age-matched healthy subjects served as controls. Liquorice ingestion elevated radial systolic (p < 0.001) and diastolic (p = 0.018) blood pressure and systemic vascular resistance (p = 0.037). During orthostatic challenge, heart rate increased less after the liquorice versus control diet (p = 0.003) and low frequency power of heart rate variability decreased within the liquorice group (p = 0.034). Liquorice intake increased central pulse pressure (p < 0.001) and augmentation index (p = 0.002) supine and upright, but in the upright position the elevation of augmentation index was accentuated (p = 0.007). Liquorice diet also increased extracellular fluid volume (p = 0.024) and aortic to popliteal pulse wave velocity (p = 0.027), and aortic characteristic impedance in the upright position (p = 0.002). To conclude, in addition to increased extracellular fluid volume and large arterial stiffness, two weeks of liquorice ingestion elevated systemic vascular resistance and augmentation index. Measurements performed at rest may underestimate the haemodynamic effects of liquorice ingestion, as enhanced central wave reflection and reduced chronotropic response were especially observed in the upright position.

## Introduction

The mineralocorticoid receptor (MR)^1^ and the glucocorticoid receptor (GR)^2^ play an important role in the regulation of blood pressure (BP). Both MR and GR are expressed in several tissues important to BP homeostasis, including the kidney, vascular wall, central nervous system, and the heart^2–4^. Cortisol binds to both GR and MR, although aldosterone is the only physiologic agonist of the MR^3^. In aldosterone target tissues the enzyme 11β-hydroxysteroid dehydrogenase type 2 (11β-HSD2) prevents cortisol from binding to the MR by inactivating it to cortisone^5^. The enzyme 11β-HSD2 may be involved in the pathogenesis of hypertension, since decreased 11β-HSD2 activity increases the activation of the MR and GR by cortisol^5^.

The elevation of BP after liquorice ingestion is well-known^6–8^. The active metabolite in liquorice, glycyrrhetinic acid (GA) that resembles the structure of cortisone, inhibits the enzyme 11β-HSD2^6^. In the kidney, 11β-HSD2 is expressed in the distal nephron, where MR activation by cortisol promotes sodium reabsorption and potassium excretion into the urine^5^. GA may also inhibit the hepatic steroid-metabolizing enzymes 5β-reductase and 3β-hydroxysteroid dehydrogenase, resulting in suppressed aldosterone catabolism^9^. These mechanisms lead to increased BP, sodium and water retention, decreased plasma potassium and aldosterone concentration, and decreased plasma renin activity^6^.

The vascular wall (smooth muscle and endothelial cells)^10^, the heart^4^ and the brain^11^ are also expression sites of the enzyme 11β-HSD2. In the vascular wall, 11β-HSD2 inhibition increases arterial tone through enhanced contractile responses to pressor hormones and decreased production of endothelial nitric oxide^12, 13^. In smooth muscle cells MR activation may contribute to vascular stiffening via remodelling of the vascular wall^1^. In the heart, glucocorticoids have been suggested to induce direct effects through MR activation^14^. Further, the infusion of GA into the lateral ventricle of the rat brain elevates BP without affecting salt or water homeostasis, suggesting a direct influence on the central nervous system^15^.

We recently reported that daily liquorice consumption in normotensive subjects for two weeks elevated peripheral and central systolic and diastolic BP, increased extracellular fluid volume, and amplified pressure wave reflection from the peripheral arterial tree^8^. However, increased peripheral arterial resistance and large arterial stiffness, and changes in cardiac function and autonomic tone could also contribute to the elevation of BP following liquorice ingestion. Since the upright cardiovascular influences of liquorice exposure are also unknown, in the present study we investigated the haemodynamic changes in healthy subjects during orthostatic challenge following voluntary liquorice intake. The balance of cardiac autonomic tone was evaluated by the use of power spectral analysis of heart rate variability (HRV)^16^.

## Methods

### Ethical statement

The investigation was performed with the understanding and written informed consent of each individual, and was approved by the Ethics Committee of the Tampere University Hospital (study code R07053M) conforming to the principles outlined in the Declaration of Helsinki. The study is registered in the database of clinical trials (ClinicalTrials.gov, ID: NCT01742702, date of registration 29 of November 2012), and is a part of an investigation on noninvasive recording of hemodynamics (DYNAMIC-study; EudraCT-number 2006-002065-39).

### Study subjects

The study population comprised of 52 healthy, normotensive (average brachial office BP 117/71 mmHg) individuals aged between 21 and 58 years. Exclusion criteria were office BP >140/90 mmHg, cardiovascular disease with regular medication, pregnancy, and consumption of liquorice >300 grams per week. Subject recruitment and data collection have been described previously^8^. The liquorice group consisted of 22 subjects (14 women and 8 men), and the aged-matched control group of 30 subjects (17 women and 13 men). When compared with our previous report focused on haemodynamics at rest^8^, the present analyses included data from two additional subjects in the liquorice group not included in the previous study.

As previously reported^8^, the regular medications in the liquorice group were 160 µg budesonide + 4.5 µg formoterol twice daily for asthma (n = 1), 5 mg escitalopram once daily for depression (n = 1), postmenopausal oestrogen replacement therapy (n = 1) and oral contraceptives (n = 5), while in the control diet group 5 female subjects used oral contraceptives and 3 had hormonal intrauterine devices. Four subjects were current smokers and 1 was previous smoker in the liquorice group, and 3 subjects were current and 4 previous smokers in the control group.

### Design

The study design has been described in detail elsewhere^8^. Briefly, this was an open-label study but subjects in the control group were not aware of acting as controls for the liquorice group. In the liquorice group, the subjects consumed commercial liquorice products (Halva liquorice^TM^ or Kouvola liquorice^TM^) daily for two weeks. Prior to baseline measurements, liquorice-containing products were not allowed for 3 weeks. During the intervention the ingested dose of liquorice was 120–300 g/d depending on the glycyrrhizin concentration of the product and the estimated dose of glycyrrhizin ranged 290–370 mg/d. All subjects maintained the liquorice diet for two weeks. In the control group, the subjects were asked to maintain their habitual diet, and the reported frequency of liquorice consumption in each subject was once per month or lower.

Physical and laboratory examinations were performed to all participants to ensure the suitability for the study, and structured questionnaires were utilized to review the lifestyle habits and medical and family history. At baseline blood and urine samples were drawn after about 12 hours of fasting with the exception that samples were not received from one subject in the control group^8^. Hemodynamic measurements were performed before and after 2 weeks of liquorice ingestion or 1–3 weeks of control diet. As exceptions, in the control diet group three subjects had consecutive measurements within 2–4 days and two subjects within approximately 4 weeks.

### Laboratory analyses

Standard 12-lead electrocardiograms were recorded with MAC5000 (GE Healthcare, Chalfont St. Giles, UK), and the recordings were normal in all subjects^8^. Plasma sodium, potassium, creatinine, glucose, cholesterol lipoproteins were determined by Cobas Integra 700/800 (F. Hoffmann-LaRoche Ltd, Basel, Switzerland), and blood cell counts by ADVIA 120 or 2120 (Bayer Health Care, Tarrytown, NY, USA).

### Haemodynamic measurement protocol

Preceding the haemodynamic recordings, caffeine containing products, smoking and heavy meal for at least 4 hours, and alcohol for at least 24 hours were to be avoided. The measurement protocol was conducted in a quiet, temperature-controlled laboratory by research nurses^17^. The impedance cardiography electrodes were placed on body surface, the tonometric sensor for radial BP on the left wrist, and a brachial cuff for BP calibration to the right upper arm^18^. The extended left arm was placed on an arm support at the level of the heart in supine and upright positions. The haemodynamics were recorded continuously for 5 minutes in supine position and for 5 minutes during orthostatic challenge to 60 degrees^17^. For the statistical analyses, mean values of each minute of the 10-min recordings were calculated.

### Pulse wave analysis

Radial BP and pulse wave form were continuously captured by a tonometric sensor (Colin BP-508T, Colin Medical Instruments Corp., USA). The radial BP recordings were calibrated twice during both 5-minute periods using contralateral brachial BP measurements. Aortic BP, pulse pressure (PP) and augmentation index (AIx) (augmentation pressure/PP*100) were determined using the SphygmoCor PWMx pulse wave monitoring system (Atcor Medical, Australia)^19^.

### Whole-body impedance cardiography

A whole-body impedance cardiography device (CircMon^R^, JR Medical Ltd., Tallinn, Estonia), which records the changes in body electrical impedance during cardiac cycles, was used to determine beat-to-beat heart rate (HR), stroke volume (SV), cardiac index (cardiac output/body surface area), pulse wave velocity (PWV), and extracellular water (ECW) volume^20, 21^. The method description and electrode configuration have been previously reported^20, 21^. Systemic vascular resistance index (systemic vascular resistance/body surface area) (SVRI) was calculated from the radial BP signal and cardiac index measured by CircMon^R^. Aortic characteristic impedance was calculated as follows: (central forward wave amplitude*ventricular ejection duration)/(2*stroke volume)^22^.

With the CircMon^R^ whole-body impedance cardiography method, the recorded PWV values show excellent correlation with values measured using ultrasound or the tonometric SphygmoCor method^18, 20^, the SV shows good correlation with 3-dimensional echocardiography recordings^23^, cardiac output values are in good agreement with values measured by the thermodilution method^21^, and the reproducibility and repeatability of the measurements are good^17, 23^.

### Frequency domain analysis of heart rate variability

The electrocardiograms recorded by the CircMon^R^ device (sampling rate 200 Hz), were analyzed using Matlab software (MathWorks Inc., Natick, Massachusetts, USA). Normal R-R intervals were recognized, and a beat was considered ectopic if the interval differed over 20% from the previous values. The artifacts were processed using the cubic spline interpolation method. The frequency domain variables were calculated using the Fast Fourier Transformation method: i) power in low frequency (LF) range (0.04–0.15 Hz), ii) power in high frequency (HF) range (0.15–0.40 Hz), and iii) LF/HF ratio^24^.

### Statistical analyses

A minimum sample size of 17 experimental and 26 control subjects was required to detect a 9 mmHg difference in the change in systolic BP from baseline with a standard deviation (SD) of 10, α-level of 0.05, and power of 80%^8^. Statistical analyses were conducted using IBM SPSS Statistics Version 24 (IBM Corporation, Armonk, NY, USA). Normally distributed data was given as means with SD, standard error of the mean or 95% confidence interval. LF and HF power were transformed to natural logarithm before analyses to yield normal non-skewed distributions. The homogeneity of variances was tested with the Levene’s test. The mean haemodynamic values were calculated from the minutes 3–5 of the recordings during supine and upright positions when the signal was most stable, and the values from the minutes 1–5 min were used for analyses of LF and HF power. Independent samples t-test was used to compare baseline data and changes between the groups. Analysis of variance for repeated measurements was applied to study interaction between time and group, and differences between the groups and over time in haemodynamic variables and HRV during rest and orthostatic challenge. In case of multiple comparisons, the results were adjusted with the Bonferroni correction, as appropriate. Chi-square test was applied to test non-continuous variables, and Spearman’s correlations (r_S_) were calculated, as appropriate. *P* < 0.05 was considered statistically significant.

### Data availability

The datasets generated during and analysed during the current study are not publicly available as our clinical database contains several indirect identifiers and the informed consent obtained does not allow publication of individual patient data. However, the datasets are available from the corresponding author on reasonable request.

## Results

The baseline characteristics of the study subjects are presented in Table 1. The demographic data, routine laboratory values, and ECW volumes did not differ between the groups.

**Table 1:** Demographic data and laboratory values at baseline.

	Control (n = 29–30*)		Liquorice (n = 22)		*P* value
	Mean	SD	Mean	SD	
Female/Male	17/13		14/8		0.776
Age (years)	33.8	8.1	34.9	9.2	0.656
Body mass index (kg/m^2^)	23.0	2.7	23.3	1.9	0.643
Waist circumference (cm)					
Male	86.9	4.4	82.6	7.1	0.100
Female	78.5	10.3	76.3	7.4	0.521
Extracellular water (l)	12.8	1.2	12.3	1.5	0.228
Hematocrit (%)	0.42	0.05	0.40	0.03	0.222
Fasting plasma					
Cholesterol (mmol/l)	4.4	0.8	4.6	0.8	0.366
Triglycerides (mmol/l)	0.94	0.49	0.83	0.32	0.374
High density lipoprotein (mmol/l)	1.72	0.39	1.83	0.36	0.305
Low density lipoprotein (mmol/l)	2.2	0.7	2.4	0.6	0.478
Glucose (mmol/l)	5.0	0.4	5.2	0.4	0.151
Creatinine (µmol/l)	76	11	77	17	0.805
Sodium (mmol/l)	140	2.0	140	1.3	0.957
Potassium (mmol/l)	3.9	0.2	3.9	0.3	0.448

^*^Blood samples for fasting plasma values were not obtained from one subject.

The *P*-values in the figures refer to the changes in haemodynamic variables induced by the liquorice diet versus controls from week 0 to week 2. The other statistical results are given below in the text.

### Haemodynamic variables at baseline

**Figure 1:**
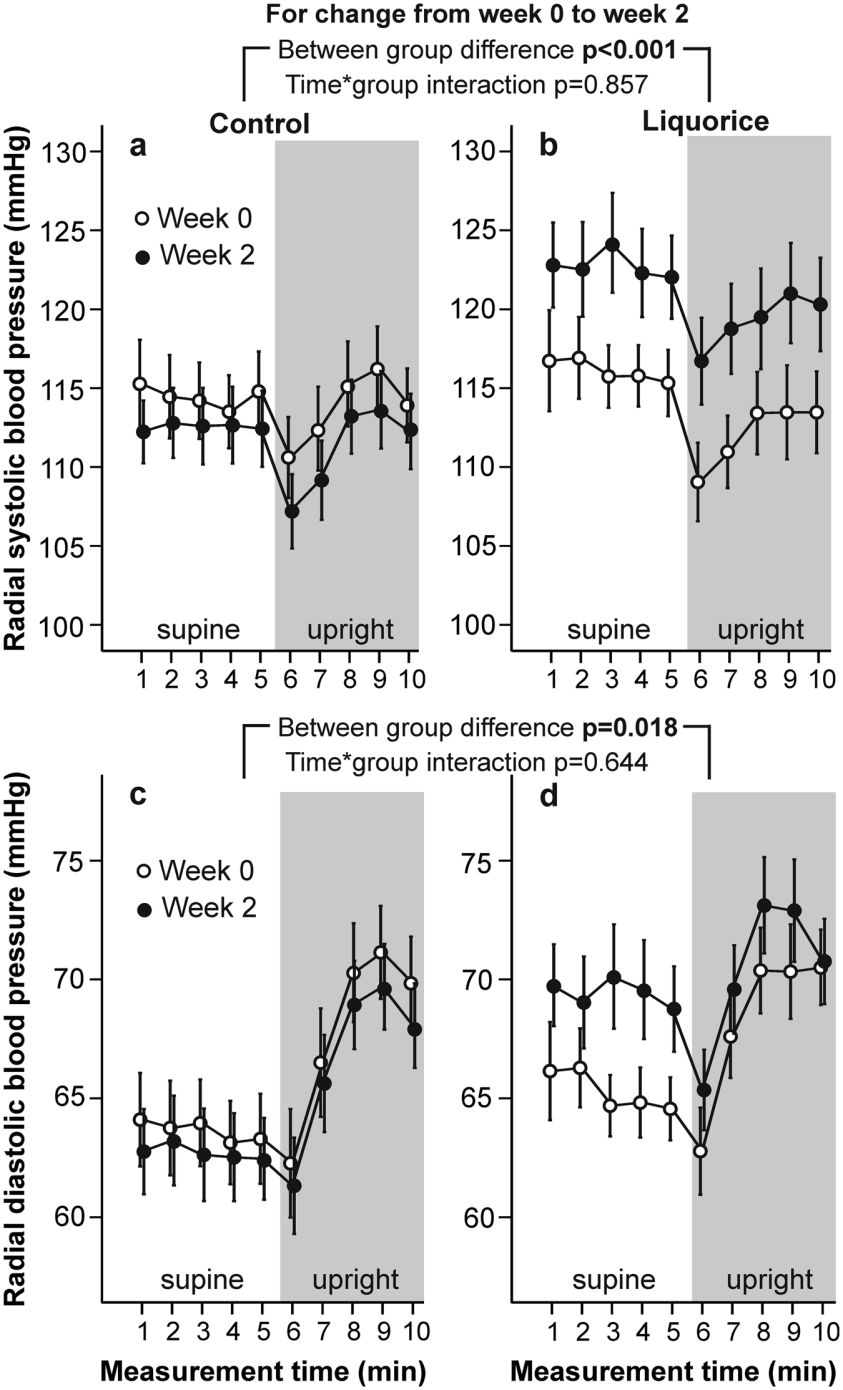
Radial systolic blood pressure (**a,b**) and diastolic blood pressure (**c,d**) before and after two weeks of control and liquorice diet. Haemodynamic data was captured continuously during the 10-min recordings, and passive head-up tilt was performed from 5 to 10 min. The graphs depict mean and standard error of the mean, and the statistical analyses are for the changes in the liquorice group versus controls from week 0 to week 2.

**Figure 2:**
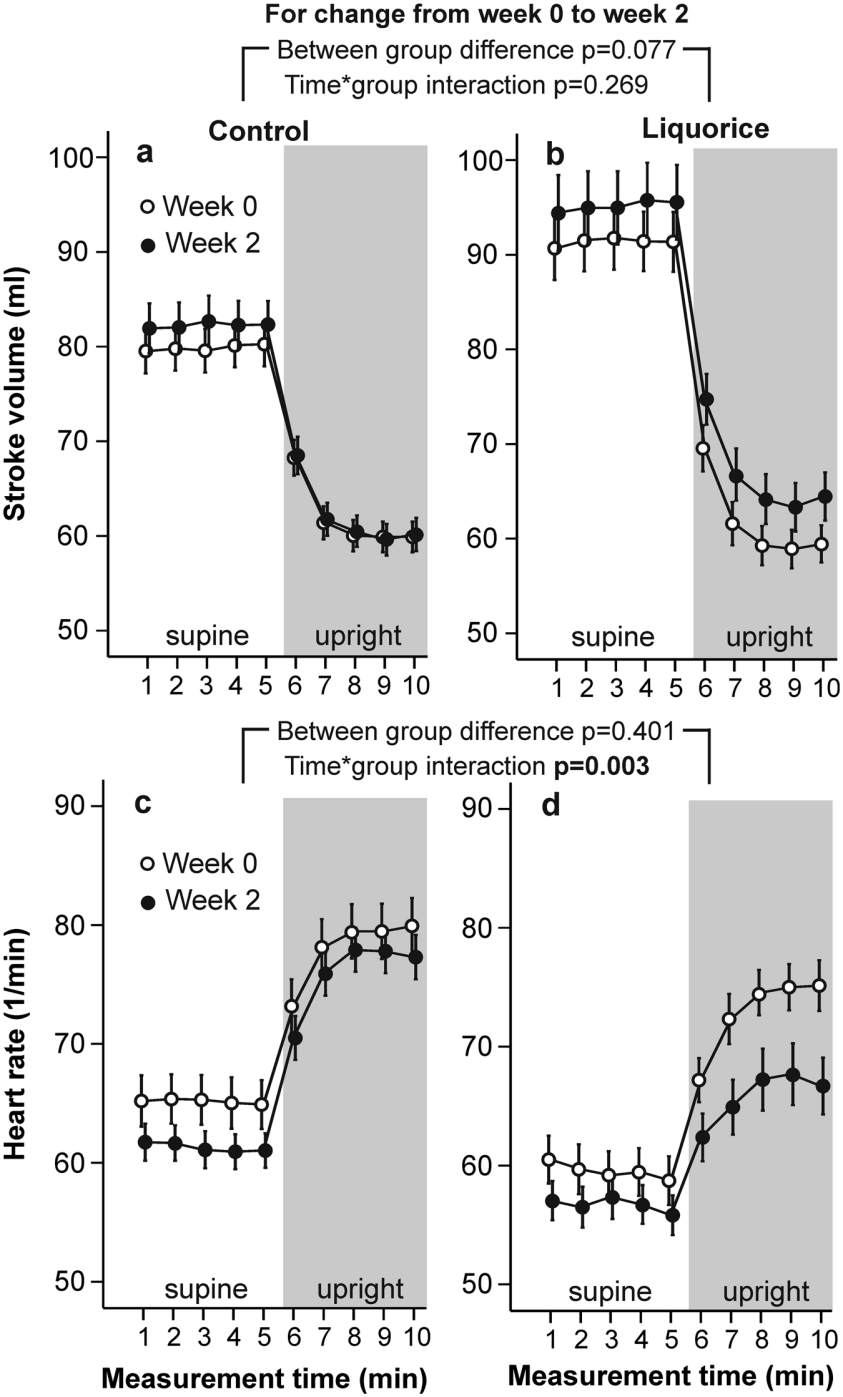
Stroke volume (**a,b**) and heart rate (**c,d**) before and after two weeks of control and liquorice diet. Mean and standard error of the mean, statistical analyses are for the changes in the liquorice group versus controls from week 0 to week 2.

**Figure 3:**
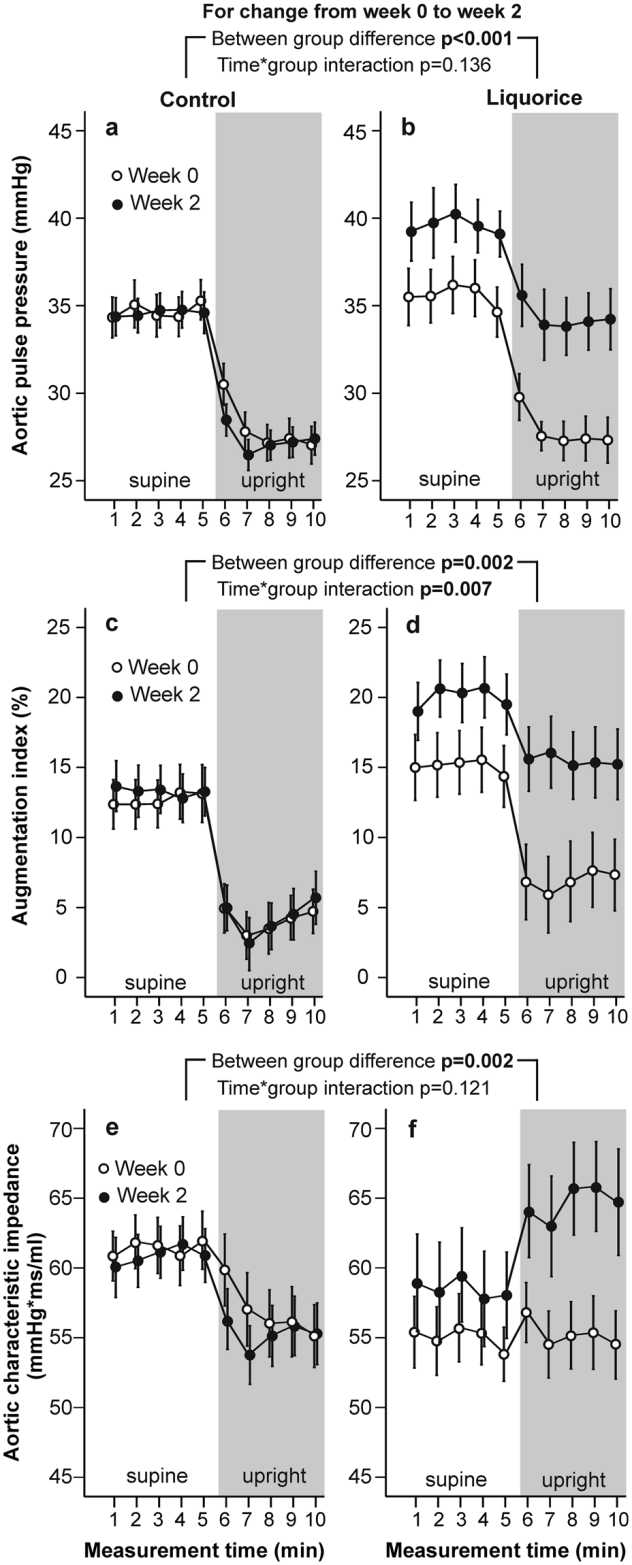
Aortic pulse pressure (**a,b**), augmentation index (**c,d**), and aortic characteristic impedance (**e,f**) before and after two weeks of control and liquorice diet. Mean and standard error of the mean, statistical analyses are for the changes in the liquorice group versus controls from week 0 to week 2.

**Figure 4:**
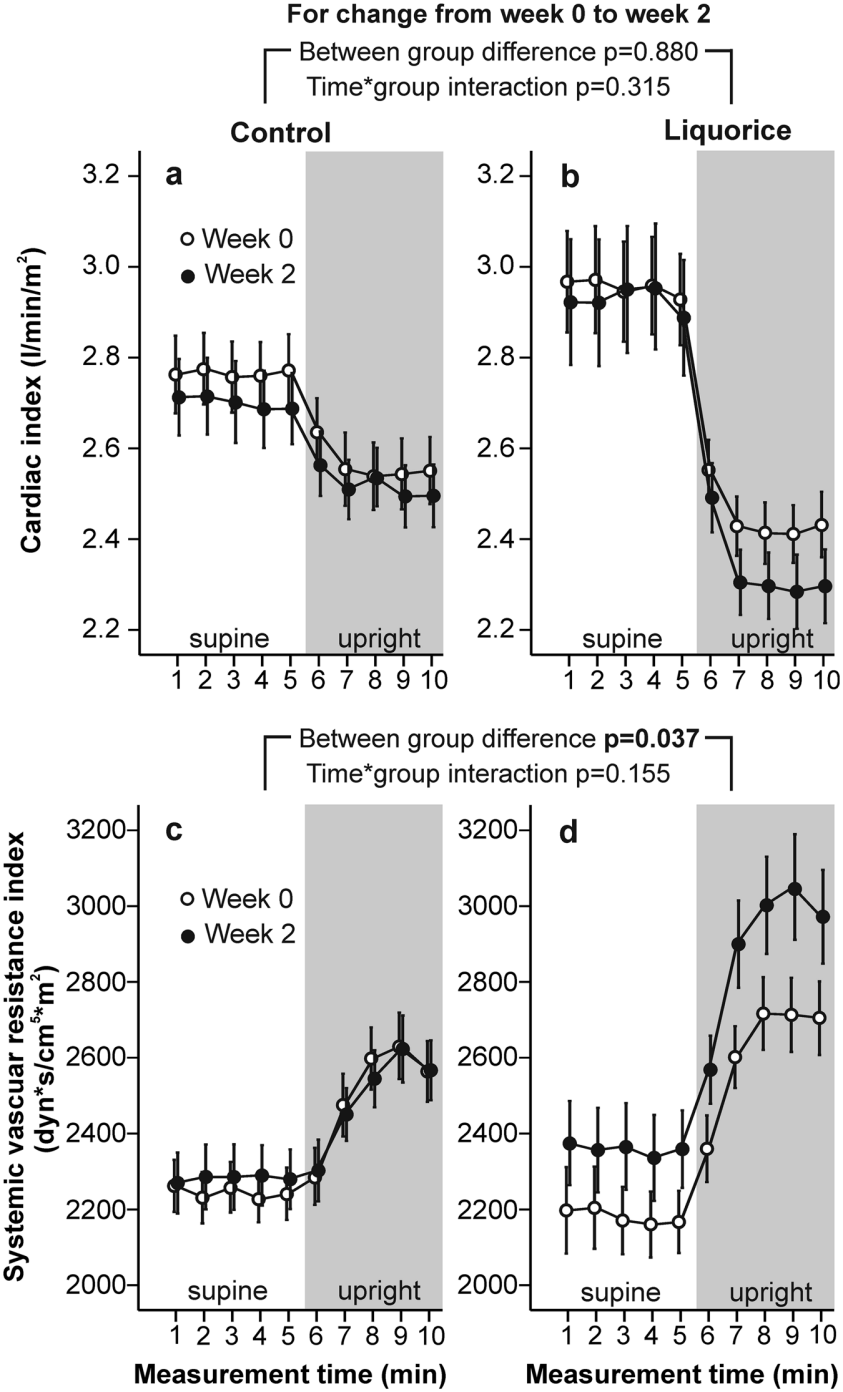
Cardiac index (**a,b**) and systolic vascular resistance index (**c,d**) before and after two weeks of control and liquorice diet. Mean and standard error of the mean, statistical analyses are for the changes in the liquorice group versus controls from week 0 to week 2.

At week 0, there were no differences in radial systolic and diastolic BP (Fig. 1), HR (Fig. 2), aortic PP, AIx and aortic characteristic impedance (Fig. 3), or SVRI (Fig. 4) during the 10-min recordings between the groups. However, analysis of variance for repeated measurements showed a significant time*group interaction (p < 0.001) in the baseline analyses of SV (Fig. 2), so that the values at rest were higher in the liquorice group than in the control group. In addition, a significant time*group interaction in cardiac index (p = 0.003) at week 0 was observed (Fig. 4) indicating a higher upright decrease of cardiac output in the liquorice group.

### Haemodynamic influences of 2-week liquorice ingestion

After two weeks of liquorice ingestion, radial systolic BP was elevated throughout the 10-min recording protocol, while an increase in diastolic BP was detected in the supine position (Fig. 1). During orthostatic challenge the increase in HR (p = 0.003) was reduced after liquorice consumption (Fig. 2). Increased aortic PP, AIx (Fig. 3), and SVRI (Fig. 4), were also observed after the liquorice diet. The elevation of AIx in the upright position was even accentuated after the liquorice diet, as indicated by the significant time*group interaction (Fig. 3, p = 0.007), and in parallel aortic characteristic impedance was increased (Fig. 3). There were no significant differences in the analyses of changes in SV (Fig. 2) or cardiac index (Fig. 4) from week 0 to 2 between the groups. Of note, the more pronounced upright decrease in cardiac index in the liquorice group persisted throughout the study (Fig. 4, p < 0.001 for the time*group interaction during the 10-min recording protocol at week 2).

The AIx is influenced by PWV, a marker of arterial stiffness, but also other factors including SVRI, HR, ventricular ejection duration, SV, and ECW volume^8, 18, 25^. Therefore, we determined the correlations between these variables in the liquorice group: the increase of AIx in the upright position significantly correlated with the changes in HR (r_S_ = −0.592, p = 0.004), SVRI (r_S_ = 0.600, p = 0.003) and ejection duration (r_S_ = 0.736, p < 0.001), but not with the changes in PWV, SV or ECW volume. Since the number of the study subjects was rather small, the use of multivariate analysis for further statistics was not feasible.

At week 2 the mean changes in ECW volume were −0.16 litres (95% CI −0.37 to 0.04) versus + 0.57 litres (95% CI −0.03 to 1.17) in the control versus liquorice groups, respectively (p = 0.024 for between group difference). The PWV mean (SD) values at baseline were 7.1 (1.1) and 7.1 (0.7) m/s in the control and liquorice groups (p = 0.880), respectively. Although the mean changes (95% CI) in PWV were small within the control and liquorice groups; −0.18 m/s (−0.38 to 0.03) versus 0.27 m/s (−0.11 to 0.65), respectively, these changes were significantly different between the groups (p = 0.027).

### Heart rate variability after 2-week liquorice ingestion

**Figure 5:**
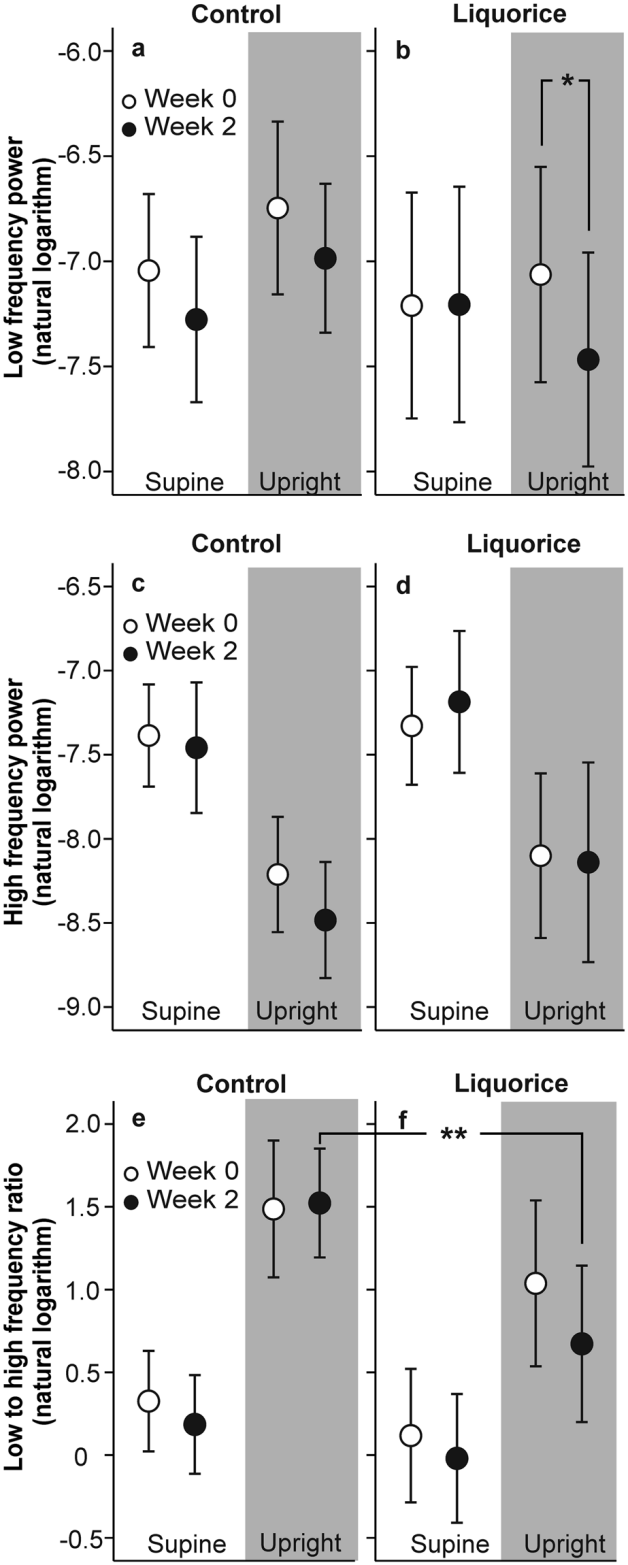
Low frequency power (**a,b**), high frequency power (**c,d**) and low to high frequency power ratio (**e,f**) before and after two weeks of control and liquorice diet in the supine and upright position. Mean and 95% confidence interval depicted, *p = 0.034 for the change within the liquorice group, **p = 0.008 for the difference in mean values at week 2 between the groups; analysis of variance for repeated measurements, post-hoc t-tests were adjusted with Bonferroni corrections for multiple comparisons.

HRV is dependent on average HR due to both physiological and mathematical reasons^26^. The mathematical dependency results from the nonlinear relationship between RR interval and HR, and as a consequence of this association the HRV analysis may be biased^26^. In order to reduce the HRV dependence on HR, we divided the LF and HF power by the average RR interval squared^26^. In the HR-corrected power spectral analyses of HRV, there were no significant within-group changes in the HF power or LF/HF ratio either in supine or upright positions (Fig. 5, p > 0.05 for all comparisons). Although there were no significant between-group differences in LF power, in the liquorice group the LF power was decreased after two weeks (Fig. 5, p = 0.034). As a minor between-group difference, the upright LF/HF ratio was lower in the liquorice group than in the control group after two study weeks (Fig. 5, p = 0.008).

## Discussion

The liquorice-induced elevation of BP has been attributed to renal sodium and water retention^6^. In line with these views, our previous findings indicated that the elevation of BP after liquorice exposure was associated with increased extracellular fluid volume^8^. In the present study, we investigated the haemodynamic influences of two-week liquorice intake during orthostatic challenge, and found that the exposure increased extracellular fluid volume and elevated radial systolic and diastolic BP, SVRI, aortic PP, and AIx. In addition, liquorice diet resulted in significant changes in PWV and aortic characteristic impedance, two indexes of aortic stiffness^22, 25^, when compared with the control group. During orthostatic challenge, liquorice ingestion resulted in a further increase of AIx indicating enhanced pressure wave reflection from the periphery, while a decreased cardiac chronotropic response was also observed. In the power spectral analyses of HRV, the LF power was decreased in the liquorice group after two weeks. Altogether, the present results indicate that the liquorice-induced elevation of BP was due to multiple mechanisms: volume overload, reduced large arterial compliance, increased peripheral vascular resistance, and enhanced pressure wave reflection from the periphery particularly in the upright position.

BP is defined as the product of cardiac output and peripheral vascular resistance^27^. The present liquorice and control groups demonstrated persistent functional differences in the regulation of cardiac output in the upright position. However, the between-group difference in cardiac index remained unchanged during the study, while SVRI was elevated after liquorice ingestion. Several mechanisms may explain the observed increase in systemic vascular resistance after the liquorice diet. Glucocorticoid activity is controlled locally and systemically by the type 1 and 2 isoforms of enzyme 11β-HSD^5^. Both 11β-HSD enzymes are expressed in the vascular wall, where they can influence vascular tone by regulating active glucocorticoid concentrations^13^. The subsequent MR activation in smooth muscle cells can result in the remodelling of the vascular wall^1^. Glucocorticoids can even potentiate the vasoconstrictor actions of angiotensin II and catecholamines in smooth muscle, and suppress vasodilatory systems including endothelial nitric oxide synthase and prostacyclin synthesis^28^. The activation of GR may reduce neuronal nitric oxide release in the arteries^29^, whereby alterations in perivascular nitrergic function may also contribute to the glucocorticoid-induced increase in vascular resistance.

Autonomic nervous system plays a significant role in the control of BP^16^. During postural challenge, a decrease in BP is sensed by the baroreceptors of carotid sinus and aortic arch, and afferent input to the nucleus of the tractus solitaries is decreased^16^. Subsequently, efferent vagal input to the sinoatrial node is reduced, sympathetic input to the heart, arterioles and venules is increased^16^, and the reduction in BP is corrected via increases in HR and systemic vascular resistance^16^. In the present study, the HR response to orthostatic challenge was attenuated after liquorice ingestion when compared with the control diet. Simultaneously, the LF power that predominantly reflects sympathetic activity^24^, was decreased in the liquorice group. Also, the LF/HF ratio, an indicator of cardiac sympathovagal balance^24^, was slightly lower in the liquorice group than in controls at week 2. The results of the power spectral analyses were in line with the observed changes in the control of HR after liquorice ingestion, and indicate that there was no increase in cardiac sympathetic tone during the intervention. Therefore, the alterations in sympathovagal balance were not the cause for the liquorice-induced elevation of BP. Previous experimental findings suggest that the changes in cardiac function could result from direct MR or GA action in the heart, as aldosterone has been found to decrease heart rate and repolarization rate in rabbit heart muscle cells^30^, while GA has been reported to show a negative inotropic action in the isolated perfused rat heart^31^.

The determination of PWV is considered the gold standard in the evaluation of arterial stiffness^25^. The present within-group changes in PWV after the liquorice and control diets were not significant, but the changes in PWV between the two groups were significantly different. We also evaluated large arterial stiffness by calculating the aortic characteristic impedance, i.e. the impedance to the left ventricular pulsatile flow^22^. Increased aortic stiffness increases the characteristic impedance and forward wave amplitude as a result from the mismatch between properties of the aortic root and peak aortic flow^22^. Although forward wave amplitude mainly represents the blood flow going forward from the heart, the results must be interpreted with caution, as wave reflection from the peripheral circulation may also influence the magnitude of forward wave amplitude^32^. We found that liquorice ingestion increased aortic characteristic impedance especially in the upright position. The present results thus suggest that already two weeks of liquorice ingestion can increase large arterial stiffness. This is probably explained by the elevation of BP, as the indices of arterial stiffness significantly depend on the prevailing BP that is distending the blood vessels^25^. It is unlikely that structural changes would take place during two weeks of liquorice exposure, but such changes are bound to result from longer periods of elevated BP^27^.

The backward pressure wave that is reflected from the branches of the arterial tree and terminations of low resistance arteries into high-resistance arterioles is characterized by the AIx^18, 25, 33^. The magnitude of AIx is influenced by arterial stiffness, but also by other factors including systemic vascular resistance, gender and height^18, 25^. In addition, AIx is reduced by approximately 4% for every 10 beats/min increase in HR^33^. In the present study, liquorice ingestion increased the AIx especially in the upright position, i.e. the upright reduction in the level of AIx was lower after liquorice exposure. This elevation of AIx during orthostatic challenge correlated with parallel changes in HR, SVRI and ejection duration, and all of these haemodynamic alterations have the potential to increase the magnitude of AIx. Of note, after liquorice withdrawal the duration of the suppressive effect of GA on 11β-HSD2 and the renin-angiotensin-aldosterone axis can be prolonged, since the normalization of increased urinary excretion of cortisol can take 2 weeks, while the recovery of suppressed plasma renin and aldosterone levels may last up to 2–4 months^6^.

The strength of the present investigation was in the detailed cardiovascular reactivity tests that enabled the detection of the liquorice-induced changes in supine and upright haemodynamics. The length of the present intervention can be considered to be adequate, since a maximal rise in BP has been demonstrated after 2 weeks of liquorice ingestion in study that also showed a linear dose-response relationship on the BP effect^7^. The present dose of glycyrrhizin (290–370 mg/day) did not exceed the daily dose of 400 mg, when the risk of adverse events has been found to increase in most individuals^34^. The study population was homogenous with normal average BMI, and the distribution of genders did not differ between the study groups, which excludes the possible confounding of the previously demonstrated differences between the sexes in the effects of liquorice ingestion^35^.

As a limitation of our study, the number of study subjects was rather small, and detailed testing of the determinants of AIx was not possible by the use of multivariate analysis. In this open-label study our aim was to test the effect of glycyrrhizin intake from liquorice on cardiovascular function, and therefore the effects of pure glycyrrhizin were not examined. A double-blinded crossover study design could not be applied, as no products with authentic liquorice taste in the absence of glycyrrhizin are available. All study subjects were instructed to maintain their habitual background diet during the study, but the stability of the dietary habits was not monitored. Of note, the participants in the control diet group were not aware of acting as controls for the liquorice study, thus among them the habitual liquorice consumption could not be unknowingly changed. Finally, the commercial liquorice product increased the intake of carbohydrates by an average of 150 grams per day corresponding the energy intake of 600 kcal per day. We have previously reported that the weight gain was 1.5 kg with simultaneous increase in ECW volume of 0.5 kg after two weeks of liquorice ingestion^8^. However, the increase in body weight did not explain the elevation of BP^8^. We have also shown that the whole-body bioimpedance PWV values correlate very well with values measured using ultrasound or the tonometric method^18, 20^.

In conclusion, this study demonstrated that in addition to extracellular fluid volume expansion, two-week liquorice exposure increased large arterial stiffness, systemic vascular resistance, and enhanced central wave reflection from the peripheral circulation. Measurements performed only at rest may result in underestimation of the haemodynamic effects of liquorice ingestion, since reduced chronotropic response and enhanced central wave reflection were especially observed in the upright position.
